# The volume–outcome relationship for hip fractures: a systematic review and meta-analysis of 2,023,469 patients

**DOI:** 10.1080/17453674.2018.1545383

**Published:** 2019-02-04

**Authors:** Eveline J A Wiegers, Charlie A Sewalt, Esmee Venema, Niels W L Schep, Jan A N Verhaar, Hester F Lingsma, Dennis Den Hartog

**Affiliations:** a Department of Public Health, Erasmus University Medical Center, Rotterdam;; b Department of Neurology, Erasmus University Medical Center, Rotterdam;; c Department of Surgery, Maasstad Hospital, Rotterdam;; d Department of Orthopaedics, Erasmus University Medical Center, Rotterdam;; e Department of Surgery-Traumatology, Erasmus University Medical Center, Rotterdam, The Netherlands

## Abstract

Background and purpose — It has been hypothesized that hospitals and surgeons with high caseloads of hip fracture patients have better outcomes, but empirical studies have reported contradictory results. This systematic review and meta-analysis evaluates the volume–outcome relationship among patients with hip fracture patients.

Methods — A search of different databases was performed up to February 2018. Selection of relevant studies, data extraction, and critical appraisal of the methodological quality was performed by 2 independent reviewers. A random-effects meta-analysis using studies with comparative cut-offs was performed to estimate the effect of hospital and surgeon volume on outcome, defined as in-hospital mortality and postoperative complications.

Results — 24 studies comprising 2,023,469 patients were included. Overall, the quality was reasonable. 11 studies reported better health outcomes in high-volume centers and 2 studies reported better health outcomes in low-volume centers. In the meta-analysis of 11 studies there was a statistically non-significant association between higher hospital volume and both lower in-hospital mortality (adjusted odds ratio (aOR) 0.87, 95% confidence interval (CI) 0.73–1.04) and fewer postoperative complications (aOR 0.87, CI 0.75–1.02). Four studies on surgeon volume were included in the meta-analysis and showed a minor association between higher surgeon volume and in-hospital mortality (aOR 0.92, CI 0.76–1.12).

Interpretation — This systematic review and meta-analysis did not find an evident effect of hospital or surgeon volume on health outcomes. Future research without volume cut-offs is needed to examine whether a true volume–outcome relationship exists.

Several studies have shown a relationship between higher surgeon or hospital volume and better health outcomes in different areas of orthopedics, such as elective hip or knee arthroplasty (Shervin et al. 2007) and in the operative treatment of scoliosis (Vitale et al. 2005).

There are several explanations for the existence of the volume–outcome relationship in surgical procedures. First, hospital staff and surgeons in particular develop more skills if they treat more patients with the same procedure. Second, hospitals with better health outcomes receive more referrals and thus increase their volume. However, studies concerning the volume–outcome relationship in hip fractures differ greatly in study design, patient population, and outcomes, which makes their results difficult to interpret.

A previous systematic review of the volume–outcome relationship in orthopedic procedures found a slight association between higher volume and mortality and postoperative complications for hip fracture patients (Malik et al. 2018[???]). However, this study included only 12 studies and did not perform a meta-analysis to estimate the size of this effect. Centralization of trauma care is of broad and current interest, which increases the volume of trauma departments in many countries. Therefore, the aim of this systematic review and meta-analysis is to evaluate and quantify the relationship between surgeon and hospital volume of hip fracture patients and health outcomes.

## Methods

For this systematic review and meta-analysis, we have used the Preferred Reporting Items for Systematic Reviews (PRISMA) guidelines and the Meta-analysis of Observational Studies in Epidemiology (MOOSE) guidelines.

### Literature search

The databases Embase.com (Medline and Embase), Web of Science, Cochrane Central and Google Scholar were searched until January 30, 2018 to identify published studies that examined the association between the volume of hip fractures and health outcomes. No time restrictions were set. The search strategy contained text words for hip fractures, hospital volume, and health outcomes and was developed by an experienced librarian (see Supplementary data). To identify additional relevant articles, reference lists of the included studies were searched. No additional databases or registries were searched.

### Inclusion and exclusion criteria

All observational cohort or cross-sectional studies that provided original data on the relationship between the volume (either hospital volume or surgeon volume) of hip fractures and health outcomes (e.g., mortality, postoperative complications, length of stay, or readmission) were eligible for inclusion in this systematic review. Studies that examined and compared levels of trauma centers were also included when numbers of patients per hospital were reported. Studies on elective arthroplasty were excluded from this systematic review. Only English-language articles and articles that were available as full text were taken into account. Conference abstracts and book chapters were excluded from our search. References of included articles were screened for potentially eligible articles.

In the meta-analyses we included articles that reported adjusted odds ratios (ORs), hazard ratios (HRs), or risk ratios (RRs) regarding the outcomes for in-hospital mortality and postoperative complications, because those outcomes were mentioned in more than 3 articles.

### Data screening and extraction

2 reviewers (CS and EW) independently screened titles and abstracts to identify potentially eligible articles. Full-text reports of such articles were retrieved and 2 reviewers (CS and EW) independently screened these full-text articles and identified eligible articles. Any disagreement was resolved through discussion or, if necessary, a third review author (HL) was consulted. The PRISMA flowchart was used to provide an overview of the data-screening process.

Study characteristics (authors, study number, publication year, study design, study period, country, data source), patient characteristics (inclusion and exclusion criteria, sample size), definition of volume (unit of measurement, continuous or categorical variable with corresponding thresholds), patient outcomes, and key findings (unadjusted and adjusted estimates) were extracted from the studies by 2 independent reviewers (CS and EW).

If we had data with multiple overlapping publications, we decided to include all relevant articles in our systematic review and only include the study with the highest quality in our meta-analysis. In case of unreported or unclear data, we attempted to contact the corresponding author for clarification.

### Quality assessment

The methodological quality of the eligible studies was independently investigated by 2 reviewers (CS and EW). Assessment of quality and generalizability of the studies was based on the key domains for observational studies (Sanderson et al. 2007). These key domains were subjectively ranked by the 2 reviewers as low or high. If any queries arose, the third reviewer (HL) was consulted. To assess potential selection bias, we examined whether inclusion and exclusion criteria were clearly described and population based. Furthermore, we assessed the methodology for measuring exposure and outcome variables. Potential design-specific sources of bias and methods to control for confounding were also examined, since these key domains are considered important when evaluating validity. For example, to check the possibility of information bias, we assessed whether studies clearly reported their cut-off of volume-groups.

### Data analysis

To assess the potential role of publication bias, a funnel plot was made. Different measures of relative risk (ORs, HRs, RRs) were considered to be equivalent since the outcomes observed could be stated to be rare. Summary estimates of the relationship between either hospital or surgeon volume and mortality or postoperative complications and their corresponding 95% confidence intervals (CI) and 95% prediction intervals (PI) were calculated with inverse variance weighted random-effects meta-analyses to account for expected heterogeneity. Effect estimates for in-hospital mortality and postoperative complications were pooled separately for hospital and surgeon volume. Studies using 30-day mortality as outcome instead of in-hospital mortality were included in the pooled effect estimates of in-hospital mortality. For the meta-analyses, different cut-offs of number of hip fracture patients per year were examined. The number of patients that was most often used to distinguish low-volume centers from high-volume centers was used to decide which studies could be included in our meta-analysis: 170 patients per year for hospital volume and 35 patients per year for surgeon volume. ORs were provided with low-volume centers set as the reference group. Effect sizes were converted when the highest-volume group was used as a reference by taking 1/OR. When adjusted effect estimates were unobtainable, we decided to include raw data. Statistical heterogeneity was assessed using the Cochran Q test quantified by the I^2^ statistic. In order to interpret the odds ratios, the baseline mortality and complication rates were calculated, weighted for the number of included patients in the meta-analysis studies. The meta-analysis was conducted with Review Manager 5.3 (https://community.cochrane.org/help/tools-and-software/revman-5).

### Funding and potential conflicts of interest

No funding sources were used for this study. No conflicts of interest were declared.

## Results

### Included studies

**Figure 1.**
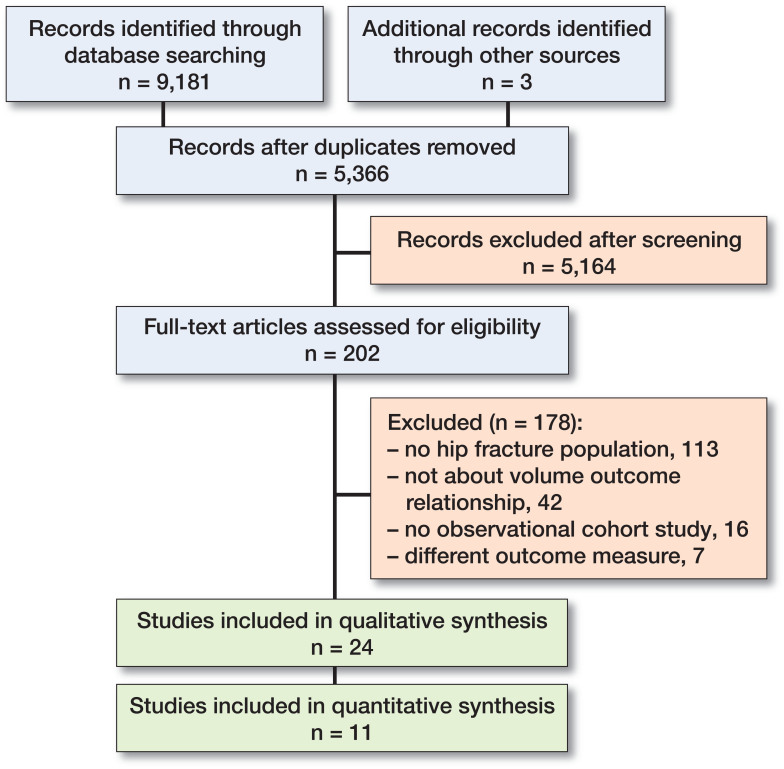
Flowchart of study selection.

The literature search identified 9,181 articles and one relevant article was identified when searching the reference lists. After removing the duplicates, 5,366 articles were screened on title and abstract (Figure 1). The remaining 202 articles were assessed full-text for eligibility, which resulted in including 24 articles in the systematic review (Flood et al. 1984, Riley and Lubitz 1985, Hughes et al. 1988, Hamilton and Hamilton 1997, Hamilton and Ho 1998, Lavernia 1998, Franzo et al. 2005, Shah et al. 2005, Genuario et al. 2008, Browne et al. 2009, Forte et al. 2010, Sund 2010, Castronuovo et al. 2011, Takahashi et al. 2011, Kristensen et al. 2014, Hentschker and Mennicken 2015, van Laarhoven et al. 2015, Elkassabany et al. 2016, Guida et al. 2016, Maceroli et al. 2016, Metcalfe et al. 2016, Nimptsch and Mansky 2017, Okike et al. 2017, Treskes et al. 2017, see Table 1, Supplementary data. References with number(s) in the following refer to numbers in Table 1). 1 author (Treskes) was contacted to provide information about the number of patients in different study groups (Treskes et al. 2017).

### Study characteristics

Of the 24 studies included, 23 studies were observational cohort studies (Table 1, Supplementary data). 21 studies (1, 3–7, 9–17, 19–24) were retrospective cohort studies, 2 studies (2, 18) were prospective cohort studies and 1 study (8) was a cross-sectional study. 12 studies (1, 3–5, 7, 12, 14–16, 18–20) were conducted in the United States, 9 studies (2, 6, 8, 11, 13, 17, 21, 23, 24) were conducted in Europe, 2 studies (9, 10) were conducted in Canada, and 1 study (22) was conducted in Japan.

Mortality was used as outcome in 23 studies (1–22, 24). In 14 studies, mortality was defined as in-hospital mortality (1, 4–7, 9–12, 14, 16, 17, 20, 24), in 7 studies as 30-day mortality (2, 3, 5, 8, 13, 15, 18), and in 2 studies as 60-day mortality (Riley and Lubitz 1985, Forte et al. 2010). 9 studies used complication rates as outcome measure (1, 7, 14–16, 18, 20, 23, 24). Typical complications assessed included postoperative infections and reoperation rates. Other outcomes reported were time to surgery, length of stay (LOS), and readmission.

21 studies evaluated hospital volume (1–13, 15–18, 20–23), 7 studies evaluated surgeon volume (1, 5, 14, 18, 19, 20, 23), and 1 study compared a level I hospital with a level II hospital (24). The cut-off used to separate high-volume centers or surgeons from low-volume centers or surgeons was highly variable. For example, 1 study used a cut-off of > 400 hip fracture patients per year (Franzo et al. 2005), while another study used ≥ 62 patients per year as a high-volume center (Shah et al. 2005).

### Quality assessment

**Figure 2.**
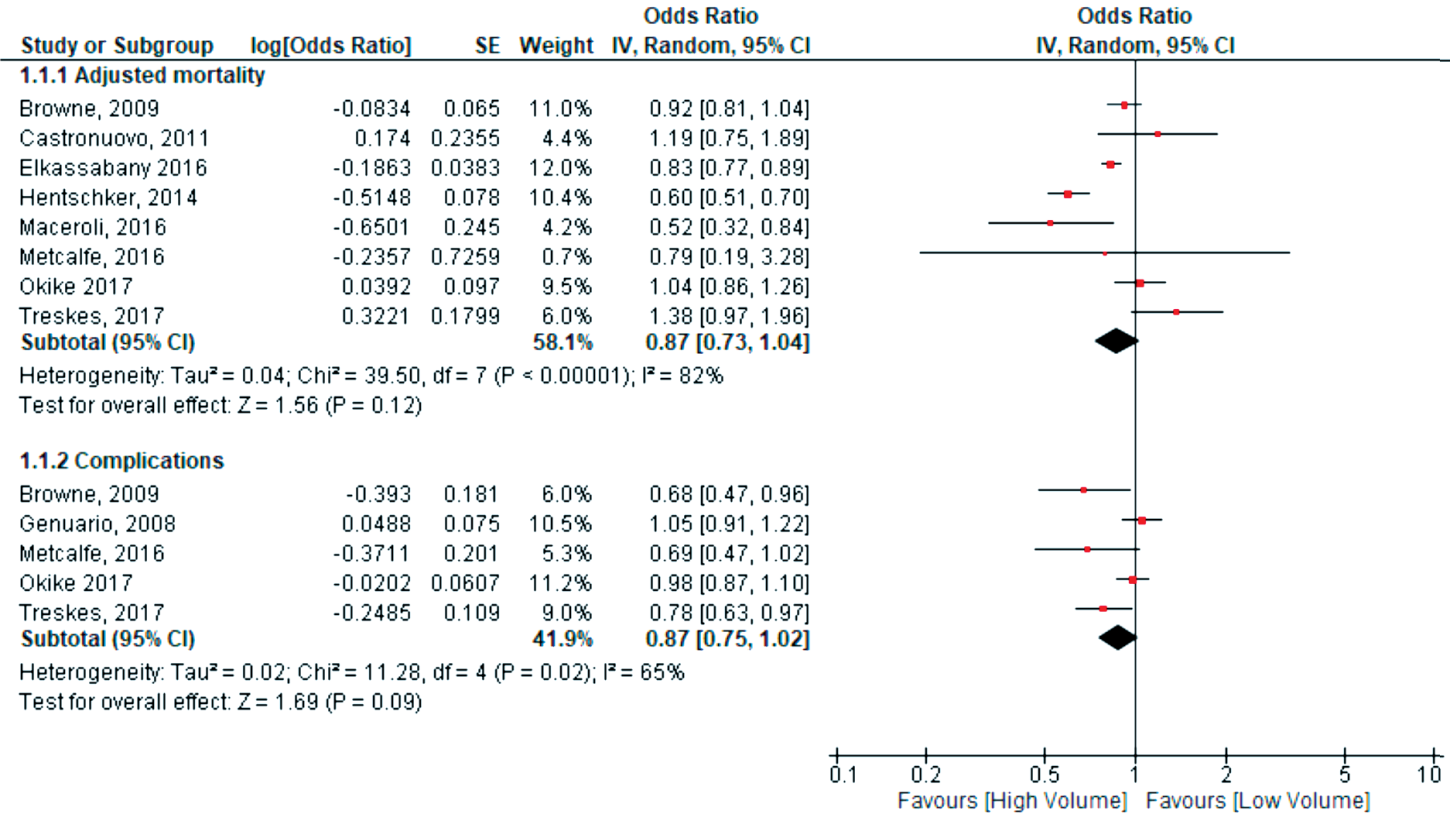
Funnel plot hospital volume. SE = standard error.

**Figure 3.**
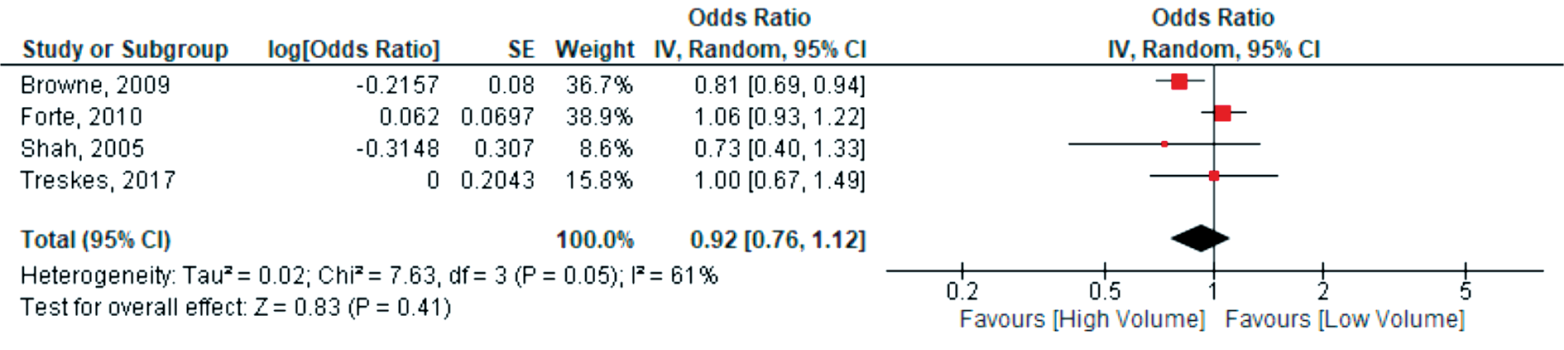
Funnel plot surgeon volume. SE = standard error.

All of the 24 included studies were population-based, and about half of the studies were nationwide (Table 2, see Supplementary data). The majority of the studies reported the number of patients per volume group, while the cut-off of the volume groups was reported in 18 studies. Adjusted odds ratios were reported in 16 studies, but crude odds ratios were reported in 3 studies. In 16 studies, adjustments for comorbidity and patients’ demographic characteristics were made. There was a limited impact of loss to follow-up in all studies. As shown in Table 2, studies that were included in our meta-analysis were considered of higher quality than studies that were excluded from our meta-analysis. These studies more often reported cut-off of volume groups and reported their results with adjustments for patient characteristics and comorbidities. The funnel plot indicated no publication bias was present (Figures 2 and 3).

### Hospital volume and mortality

20 studies assessed the relationship between hospital volume and mortality, either in-hospital or 30-day (1, 2, 4–18, 20, 21, 24). 10 studies did not find any association between hospital volume and in-hospital mortality (1, 2, 4, 8, 10, 16–18, 20, 21). 8 studies observed that high-volume centers had a statistically significantly lower mortality rate compared with low volume centers (5, 7, 9, 11, 12, 14, 15, 24). There were 2 studies which reported that higher hospital volume was associated with a higher mortality rate compared with low hospital volume (6, 13).

**Figure 4.**
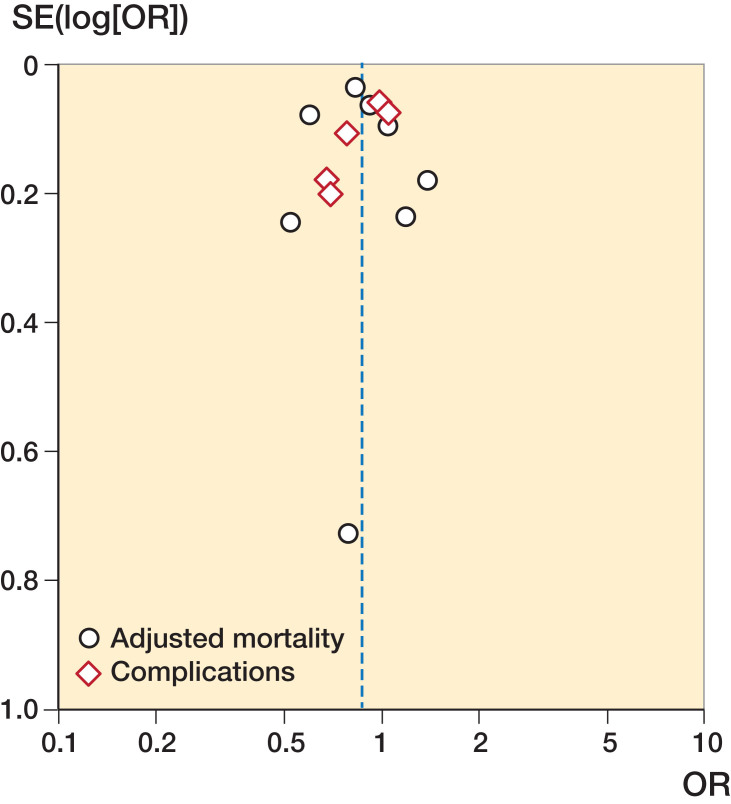
Comparisons of high- and low-volume hospitals. SE = standard error, df = degrees of freedom, and IV = inverse variance.

8 studies provided adjusted odds ratios with the corresponding cut-off of 170 patients with hip fractures per year and could be included in the meta-analysis (1, 2, 3, 11, 15, 16, 18, 23). The meta-analysis showed a trend towards higher hospital volume in terms of in-hospital mortality, although it was not statistically significant (OR 0.87, CI 0.73–1.04, 95% PI 0.51–1.49). Between-study heterogeneity was large (I^2^ = 82%) (Figure 4). The weighted baseline in-hospital mortality risk in low-volume centers for studies included in the meta-analysis was 8%.

### Hospital volume and complications

8 studies evaluated the relationship between hospital volume and complications (1, 7, 15, 16, 18, 20, 23, 24). 5 studies found significantly more complications in low-volume centers compared with high volume centers (1, 15, 20, 23, 24). 3 studies described no differences in complication rates between low- and high-volume centers (7, 16, 18).

5 studies provided adjusted odds ratios for postoperative complications (1, 7, 16, 18, 23) (Figure 4). There was a statistically non-significant relationship between higher hospital volume and postoperative complications with reasonable heterogeneity (OR 0.87, CI 0.75–1.02, I^2^ = 65%, 95% PI 0.52–1.46). The weighted baseline complication rate in low-volume centers for studies included in the meta-analysis was 16%.

### Hospital volume and length of stay

8 studies assessed the relationship between hospital volume and hospital length of stay (7, 9, 10, 12, 13, 16, 20, 22). 5 studies found a significant relationship between hospital volume and length of stay (7, 13, 16, 20, 22). 4 studies reported that low-volume centers were associated with a longer length of stay (7, 16, 20, 22), while one study (13) observed that high-volume centers had a longer length of stay compared with low-volume centers.

No meta-analysis regarding hospital volume and length of stay could be performed, since we did not include enough studies that provided adjusted ORs.

### Surgeon volume and mortality

2 studies observed a significant relationship between surgeon volume and in-hospital mortality (1, 20). Low surgeon volume was associated with a significant higher mortality rate compared with high surgeon volume in both studies. 3 studies did not find a significant relationship between surgeon volume and in-hospital mortality (5, 14, 19).

**Figure 5.**
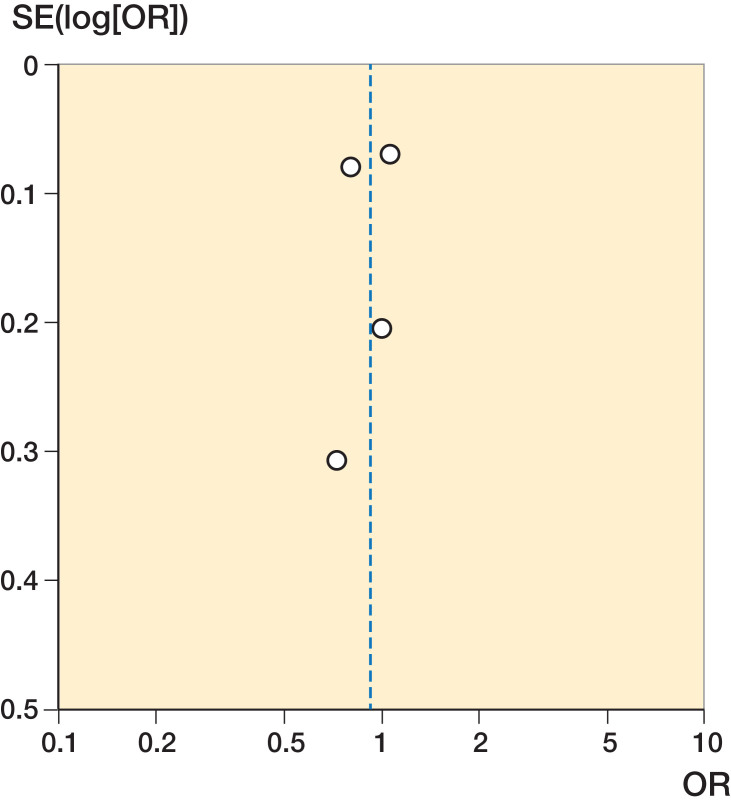
Comparisons of high- and low-volume surgeons. SE = standard error, df = degrees of freedom, and IV = inverse variance.

The only meta-analysis regarding surgeon volume was on mortality. A cut-off of 15 patients per surgeon per year was used with low volume as reference group. 4 studies provided adjusted odds ratios regarding surgeon volume and in-hospital mortality (Figure 5) (1, 5, 20, 23). There was no significant relationship between surgical volume and in-hospital mortality with moderate heterogeneity (OR 0.92, CI 0.76–1.12, I^2^ = 61%, 95% PI 0.44–1.94, p = 0.4). The weighted baseline in-hospital mortality rate for low-volume surgeons for studies included in the meta-analysis was 3.0%.

### Surgeon volume and complications

5 studies looked at the relationship between surgeon volume and complications (1, 14, 18, 20, 23). 4 studies found no statistically significant relationship between higher surgeon volume and postoperative infections and morbidity (1, 18, 20, 23). 1 study reported a statistically significant relationship between higher surgeon volume and complications comparing high surgeon volume to low surgeon volume (14).

### Surgeon volume and length of stay

3 studies evaluated the relationship between surgeon volume and length of stay. All of these studies showed that high surgeon volume was significantly associated with a lower length of stay (1, 14, 20).

## Discussion

This study included 24 studies that evaluated the volume–outcome relationship for hip fractures. There was no consistent effect of the impact of hospital and surgeon volume in terms of health outcomes. The quality of the included studies was reasonable. Nearly all studies were population based, reported the total number of severely injured patients, had limited impact of loss of follow-up, and reported crude ORs or mortality percentages. However, not all studies reported their cut-off in volume groups clearly or presented adjusted ORs. The methodological approaches of assessing the relationship between hospital and/or surgeon volume and outcomes greatly varied between studies. Therefore, we applied strict inclusion criteria for the meta-analysis to reduce heterogeneity. Our meta-analysis suggested that in-hospital mortality and postoperative complications are lower in high-volume hospitals (defined as > 170 cases per year), although this relationship was not statistically significant. Also, there was no association between surgeon volume (high volume defined as > 35 cases per year) and in-hospital mortality.

### Definition of volume

The definition of either hospital or surgeon volume is heterogeneous and in most studies an arbitrarily chosen cut-off was used. In 1 study, 62 annual cases were considered as high hospital volume (Shah et al. 2005), while this number was considered as low hospital volume in another study (Kristensen et al. 2014). For this reason, only a few studies where comparable cut-offs were used could be included in the meta-analysis.

To evaluate whether the lack of a clear relationship is a real effect, it is important not to use arbitrarily selected volumes but to treat volume as a continuous variable. This makes it possible to identify whether a true volume–outcome relationship exists and decreases the information loss due to categorizing (MacCallum et al. 2002).

### Case-mix differences

It is known that large case-mix differences exist between high- and low-volume centers. For example, Level I trauma centers with a high volume of patients admit patients who have more complex fractures and higher comorbidity then Level II trauma centers (Cudnik et al. 2009). These differences may bias outcomes as patients are not randomly distributed across hospitals.

Some studies clearly reported confounders used in adjustments, but most of them were insufficient to completely adjust for the case-mix differences. For instance, demographic factors and comorbidity were not always taken into account because this information was unavailable. All studies that we included in our meta-analysis did adjust for patient demographic characteristics and comorbidity.

### Underlying mechanism volume–outcome relationship

It is important to investigate which underlying mechanism drives the volume–outcome relationship. The “practice makes perfect theory,” which implies that repetition of a certain procedure is associated with a better outcome, might be a good explanation for studies that report high-volume hospitals as performing better. However, this “practice makes perfect theory” cannot be fully responsible for reported volume–outcome relationships.

Several potential factors of processes of care might be behind the possible effect of volume. For example, some low-volume hospitals have longer delays from admission to surgery, which might contribute to an increased mortality rate (Forte et al. 2010). In addition, it has been suggested that high-volume hospitals might have more developed pathways, processes like a reduced delay to the operating theater, and better access to alternative forms of anesthesia (Metcalfe et al. 2016). High-volume hospitals may have more established screening systems that elevate the complication rates in higher volume centers, but improve patient outcomes in the long term (Genuario et al. 2008). Furthermore, in high-volume centers physicians could often more easily make an appeal to senior doctors or physicians specialized in orthogeriatrics, which might prevent the development of complications such as postoperative delirium.

On the other hand, mechanisms that are in favor of low-volume hospitals are proposed as well. As indicated by a previous study, the effect of volume decreased: those with 350 hip fracture patients or more had higher mortality rates. First, high-volume hospitals less frequently use guidelines on recommended processes of care, which might be a key mediator at high-volume hospitals. Second, it is speculated that hip fracture patients experience less attention from nurses in high-volume hospitals since they would have to “compete” with more complicated orthopedic surgery patients, although evidence for this mechanism does not exist (Kristensen et al. 2014).

The surgeon volume relationship is expected to be influenced by several factors. The selection of the appropriate procedure and intraoperative technique might differ due to local standards or guidelines. Furthermore, surgical outcome may also be influenced by preoperative planning and postoperative care (Browne et al. 2009), which highly depends on the workflow in a hospital. Most studies did only focus either on the number of cases per year per surgeon or the number of cases per year per hospital without accounting for the overall experience of the individual surgeon. In order to gain a better understanding of the interaction among experience, surgeon, and hospital volume, future research should account for surgeon experience.

Since there are also studies that did not find a positive or a negative association between volume and patient outcomes, we expect other factors that are not related to volume to play a role in the outcome of hip fracture patients as well. Improvements in surgical and anesthetic techniques or an extended application of thromboembolism and hospital infection prevention protocols might be alternative explanations for reductions of mortality risk and postoperative complications (Franzo et al. 2005). Most studies included only patients who underwent arthroplasties (both hemi- and total hip). However, some studies also included patients who underwent open reduction or internal fixation or both. This might possibly have influenced the results, since high-volume centers or surgeons might more easily choose to do more complicated surgery. Further research is needed to assess the importance of these factors compared with hospital and or surgeon volume.

### Limitations

In addition to the bias caused by case-mix differences, our study could also suffer from publication bias. Studies showing no volume–outcome relationship, or an inverse volume–outcome relationship might be unpublished. But, as the funnel plot shows no correlation between effect size and their standard error, publication bias seems unlikely. Furthermore, mortality, complications, and length of stay might not be sensitive enough to detect outcome differences after hip fractures. Other quality indicators such as operation time or quality of life might be more influenced by hospital or surgeon volume than hard outcomes such as mortality.

All included studies in our meta-analysis used mortality as outcome; however, variability in the time-point of mortality and volume cut-offs is a likely source of heterogeneity and a potential limitation of our study. Nevertheless, substantial systematic differences are unlikely to be present since different time-points of mortality could still indicate the effect of volume on outcomes. Furthermore, we used a random effects model to account for between-study heterogeneity.

In summary, this systematic review and meta-analysis did not find an evident effect of either hospital or surgeon volume on different health outcomes. Studies examining the volume–outcome relationship in patients with hip fracture appear to be heterogeneous, specifically the cut-offs that are used. Future research without volume cut-offs is needed to examine whether a true volume–outcome relationship exists, to assess the best cut-off for high volume and determine which processes of care are important in the care of patients with hip fracture.

### Supplementary data

Tables 1–2 and the search strategy are available as supplementary data in the online version of this article, http://dx.doi.org/ 10.1080/17453674.2018.1545383

## Supplementary Material

Supplemental Material

## Notes

The authors would like to thank Wichor Bramer, biomedical information specialist from Erasmus Medical Center, for his help with the search strategy. Also, they would like to thank Kaij Treskes (PhD student, Academic Medical Center Amsterdam) for providing data for the meta-analysis.

EW/CS: literature search, study selection, data extraction, data analysis, drafting the manuscript—contributed equally. EV/HL: study selection, data-analysis, critical revision of the manuscript. NS/JV: critical revision of the manuscript.DDH: study selection, critical revision of the manuscript.

*Acta* thanks Michael Brix and Pia Kjaer Kristensen for help with peer review of this study.
