# Supplementary material for: The volume–outcome relationship for hip fractures: a systematic review and meta-analysis of 2,023,469 patients
Source: Acta Orthop. 2019 Feb 4;90(1):26–32. doi: 10.1080/17453674.2018.1545383 (PMC6366538; doi:10.1080/17453674.2018.1545383)
Supplement: Supplemental Material [file IORT_A_1545383_SM9810.pdf]

Supplementary data

Table 1. Study characteristics

Number, author, year • Key findings	Study design	Country	Period	Data source	Patients	Unit	Low	High	Outcomes	Reported data
1. Browne, 2009	RCS	USA	1998–2002	NIHS	97,894	Hospital	< 57	> 132	In-hospital mortality, complications	OR
• Higher hospital volume is not associated with mortality, but it is with nonfatal morbidity and length of stay +/–										
						Surgeon	< 7	> 15	Some as above	OR
• Higher surgeon volume is associated with decreased mortality +										
2. Castronuovo, 2011	PCS	Italy	2006	RHD	6,896	Hospital	≤ 45	> 200	30-day mortality	HR
• Higher hospital volume is not associated with mortality –										
3. Elkassabany, 2016	RCS	USA	2007–2009	RHD	458,526	Hospital	< 13	> 175	Readmission to an acute care hospital for any cause within 30 days, death at 30 days, readmission at 30 days	OR
• Higher case volumes are associated with lower rates of readmission and mortality +										
4. Flood, 1984	PCS	USA	1972	CPHA	52,368	Hospital	≤ 44.8	> 44.8	In-hospital mortality	SMR
• No association between hospital volume and mortality –										
5. Forte, 2010	PCS	USA	2000–2002	MPA	192,365	Hospital	1–17	≥ 79	In-hospital mortality, 30/60/90-day mortality	RR
• Only the highest volume hospitals showed an inpatient mortality benefit +										
						Surgeon	1–4	≥ 18	In-hospital mortality, 30/60/90-day mortality	RR
• Surgeons in the second quartile, just below the median, had the highest mortality rates –										
6. Franzo, 2005	RCS	Italy	1996–2000	DR	6,629	Hospital	< 45	> 400	In-hospital mortality, 6 months, 1 year mortality	OR
• High hospital volume was associated with worse outcomes + (inverse)										
7. Genuario, 2008	RCS	USA	1997–2003	NTDB	9368	Hospital	1–23	> 60	In-hospital mortality, complications, LOS	OR
• Moderate volume had the lowest odds of death, LOS was shortest in high volume, complication rates were similar +/–										
8. Guida, 2016	CSS	Italy	2014	NOE	69,134	Hospital	–	–	(continuous) 30-day mortality	Spearman
• No association between hospital volume and mortality –										
9. Hamilton, 1997	PCS	Canada	1991–1993	DR	7383	Hospital	< 34	> 71	In-hospital mortality, LOS	MR
• Higher volume is associated with higher probability to live +										
10. Hamilton, 1998	PCS	Canada	1990–1993	DR	7483	Hospital	≤ 32	≥ 73	In-hospital mortality, LOS	MR
• Hospitals performing more surgeries experienced no significant improvements in outcomes –										
11. Hentschker, 2015	PCS	Germany	2007	DR	7980	Hospital	10–58	151–387	In-hospital mortality	OR
• Higher case volumes have on average a significant lower probability to die +										
12. Hughes, 1988	PCS	USA	1982	CPHA	44,905	Hospital	–	–	(continuous) In-hospital mortality, LOS	Coefficient
• Mutual causality between volume of hip fracture patients and outcomes +										
13. Kristensen, 2014	PCS	Denmark	2011–2011	DMHFR	12,065	Hospital	≤ 151	≥ 351	30-day mortality, time to surgery, LOS, quality indicators	OR
• Patients admitted to high-volume centers had higher mortality rates, lower quality and longer LOS + (inverse)										
14. Lavernia, 1998	PCS	USA	1992	DR	5,604	Surgeon	≤ 10	> 30	In-hospital mortality, LOS, complications	p-value
• Higher surgeon volume was significantly associated with LOS +										
15. Maceroli, 2016	RCS	USA	2000–2010	SPARCS	3,986	Hospital	< 47	> 171	30-day/1-year mortality, 90-day complication rate	HR
• High-volume centers are associated with lower mortality and complication rates +										
16. Metcalfe, 2016 16	RCS	USA	2007–2011	DR	91,401	Hospital	< 20	> 215	In-hospital mortality, LOS, complications	OR
• Time to operation and LOS were prolonged in low-volume centers, no differences in clinical outcomes +/–										
17. Nimptsch, 2018	RCS	Germany	2009–2014	DR	710,962	Hospital	< 64	≥ 283	In-hospital mortality	OR
• No relation between volume and outcome observed –										

Table 1 continued

Number, author, year • Key findings	Study design	Country	Period	Data source	Patients	Unit	Low	High	Outcomes	Reported data
18. Okike, 2017	PCS	USA	2010– 2013	KPHFR	14,294	Hospital	< 125	≥ 187	30-, 90-, 1-year mortality, postoperative morbidity (lifetime reoperation, 90-day medical complications, and 30-day unplanned readmission)	OR
• Hospital volume did not influence observed rates of mortality and postoperative morbidity –										
						Surgeon	< 14	≥ 62	Some as above	OR
• Mortality and postoperative morbidity rates did not differ by surgeon volume –										
19. Riley, 1985	PCS	USA	1979– 1980	HCFA	20,161	Surgeon	< 4	> 9	60-day mortality	Coefficients
• The results indicate an association between higher surgeon volume and mortality +										
20. Shah, 2005	PCS	USA	1988– 2000	NIHS	173,508	Hospital	1–19	≥ 62	In-hospital mortality, complication rates, LOS	OR
• Low-volume hospitals were associated with prolonged LOS and higher complication rates +										
						Surgeon	1–3	≥ 12	Same as above	OR
• Low-volume surgeons were associated with higher mortality and prolonged LOS +										
21. Sund, 2010	PCS	Finland	1998– 2001	FHCR	22,857	Hospital	–	–	(continuous) 4-month mortality	Graphical
• There are no indications that patients benefit from high-volume centers –										
22. Takahashi, 2011	PCS	Japan	2006	DR	8,920	Hospital	< 28	≥ 28	Postoperative LOS	OR
• Patients treated in higher-volume hospitals have shorter length of postoperative hospital stay +										
23. Treskes, 2017	PCS	The Netherlands	2010– 2013	TR	4,552	Hospital	< 96	> 170	Complications	OR
• High hospital volume is associated with fewer operations and surgical site infections +										
						Surgeon	< 15	> 25	Complications	OR
• Surgeon volume is not associated with fewer operations and surgical site infections –										
24. Van Laarhoven, 2015	RCS	The Netherlands	2008– 2012	DR	1,629	Hospital	–	–	In-hospital mortality,c omplications, time to operative treatment	p-value
• Level II centers had shorter time to operation, shorter LOS, complication rate was lower and mortality was lower +										

Abbreviations:
CPHA = Commission of Professional Hospital Activities
CSS = cross-sectional study
DR = discharge register
FHCR = Finnish Health Care Register
HCFA = Health Care Financing Administration
KPHFR = Kaiser Permanente Hip Fracture Registry
LOS = Length of stay
MPA = Medicare provider analysis
NIHS = nationwide inpatient sample database
NOE = National Outcome Evaluation Program Edition
NTDB = National Trauma Data Bank
PCS = prospective cohort study
RCS = retrospective cohort study
RHD = regional hospital discharge
SMR = standardized mortality ratio
SPARCS = Statewide Planning and Research Cooperative System
TR = Trauma Registry

Table 2. Quality assessment

Study	A	B	C	D	E	F	G	H	I	J	K	L	M	N	O	P
1. Browne	+	+	+	+	–	+	+	+	+	–	+	+	+	+	–	–
2. Castronuovo	+	–	+	+	+	+	+	–	+	–	+	+	+	+	+	+
3. Elkassabany	+	–	+	+	–	+	+	–	+	–	+	+	+	+	+	+
4. Flood	–	–	+	+	+	–	–	–	–	–	–	–	–	–	–	+
5. Forte	+	+	+	+	–	+	+	+	+	–	+	+	+	+	–	+
6. Franzo	+	–	+	+	+	+	+	–	+	–	+	+	+	+	–	+
7. Genuario	+	+	+	+	+	+	+	–	+	+	+	+	+	+	+	+
8. Guida	+	+	+	+	–	–	–	–	–	–	–	–	–	–	–	–
9.Hamilton 1997	+	–	+	+	–	+	–	–	–	–	–	–	–	–	–	+
10. Hamilton 1998	+	–	+	+	–	+	+	–	+	–	+	+	+	+	–	+
11. Hentschker	+	+	+	+	+	+	+	–	+	–	+	+	+	+	–	+
12. Hughes	+	–	+	+	–	–	–	–	–	–	–	–	–	–	–	+
13. Kristensen	+	+	+	+	+	+	+	–	+	+	+	+	+	+	+	–
14. Lavernia	–	–	+	+	–	+	–	–	–	–	–	–	–	–	–	–
15. Maceroli	+	–	+	+	+	+	+	–	+	–	+	+	+	+	+	+
16. Metcalfe	+	–	+	+	+	+	+	–	+	–	+	+	+	+	+	+
17. Nimptsch	–	+	+	+	+	–	+	–	–	+	+	+	+	+	–	+
18. Okike	+	–	+	+	–	+	+	–	+	–	+	+	+	+	+	+
19. Riley	–	–	+	+	+	+	–	–	–	–	–	–	–	–	–	–
20. Shah	+	+	+	+	+	+	–	+	+	–	+	+	+	+	+	+
21. Sund	–	+	+	+	–	–	–	–	–	–	–	–	–	–	+	+
22. Takahasi	+	+	+	+	–	+	+	–	+	–	+	+	+	+	–	+
23. Treskes	+	–	+	+	+	+	+	+	+	–	+	+	+	+	+	+
24. Van Laarhoven	+	–	+	+	+	–	–	–	–	–	–	–	–	–	+	–

A. Inclusion and exclusion criteria clearly described?
B. Nationwide?
C. Population-based?
D. Total number of hip fracture patients reported?
E. Number of patients per volume group reported?
F. Cut-off volume groups clearly reported?
G. OR/HR hospital-volume reported?
H. OR/HR surgeon-volume reported?
I. OR/HR reported + 95% confidence intervals?
J. Crude ORs/HRs reported?
K. Adjust ORs/HRs reported?
L. All confounders used in adjusted OR/HR clearly reported?
M. Adjust for patients demographic characteristics?
N. Adjust for comorbidity?
O. No conflict of interest declared?
P. Funding sources identified?

Search strategy

Search results January 30, 2018

	Total	Removed duplicated
Embase.com	2,681	2,622
Medline Ovid	2,615	677
Web of science	2,979	1,374
Cochrane CENTRAL	706	527
Google scholar	200	163
Total	9,181	5,363

Embase.com

('high volume hospital'/exp OR 'low volume hospital'/exp OR (((volume*) NEAR/3 (high OR low OR surg* OR hospital* OR critical-patient* OR severe-patient* OR trauma-patient* OR emergen*-patient* OR centre* OR center*)) OR ((number-of) NEXT/1 (critical-patient* OR severe-patient* OR trauma-patient* OR emergen*-patient*)) OR ((high* OR low*) NEXT/1 exposure*) OR (size NEAR/3 (department* OR hospital* OR centre* OR center*)) OR caseload* OR case-load* OR (volume* NEAR/3 outcome*) OR patient-volume* OR 'volume of patients'):ab,ti) AND ('emergency'/de OR traumatology/exp OR 'emergency care'/de OR 'emergency health service'/exp OR 'emergency patient'/exp OR 'emergency ward'/exp OR 'emergency medicine'/exp OR burn/de OR 'burn unit'/de OR 'fracture'/exp OR accident/exp OR 'accidental injury'/exp OR injury/mj OR 'blunt trauma'/exp OR 'penetrating trauma'/exp OR 'crush trauma'/de OR 'multiple trauma'/de OR 'head and neck injury'/exp OR 'limb injury'/exp OR 'sport injury'/exp OR 'thorax injury'/de OR 'abdominal injury'/de OR 'nervous system injury'/exp OR ((emergen* NEAR/3 (centre* OR center* OR ward* OR patient* OR admiss* OR admitt* OR department* OR room*)) OR trauma* OR neurotrauma* OR multitrauma* OR burn* OR fracture* OR accident* OR (acute* NEAR/3 care) OR ((accidental OR blunt OR penetrating OR crush OR multiple OR head OR neck OR limb OR sport* OR athlet* OR thora* OR abdom* OR brain*) NEAR/3 injur*) OR (ED NEAR/3 patient*)):ab,ti) AND ('treatment outcome'/exp OR 'hospital admission'/exp OR 'hospital discharge'/de OR hospitalization/de OR 'mortality'/exp OR 'fatality'/exp OR 'survival'/exp OR 'length of stay'/de OR 'convalescence'/de OR (outcome* OR (admiss* NEAR/3 ('intensive care' OR icu OR icus OR hospital*)) OR mortalit* OR fatal* OR surviv* OR discharge* OR ((length OR duration* OR long* OR short*) NEAR/6 stay) OR recover* OR convalescen* OR hospitalization* OR hospitalisation* OR ((treat* OR management* OR therap*) NEAR/3 (fail* OR succes*))):ab,ti) NOT ([animals]/lim NOT [humans]/lim) NOT ([Conference Abstract]/lim OR [Letter]/lim OR [Note]/lim OR [Editorial]/lim) AND [english]/lim

Medline Ovid

("Hospitals, High-Volume"/ OR "Hospitals, Low-Volume"/ OR (((volume*) ADJ3 (high OR low OR surg* OR hospital* OR critical-patient* OR severe-patient* OR trauma-patient* OR emergen*-patient* OR centre* OR center*)) OR ((number-of) ADJ (critical-patient* OR severe-patient* OR trauma-patient* OR emergen*-patient*)) OR ((high* OR low*) ADJ exposure*) OR (size ADJ3 (department* OR hospital* OR centre* OR center*)) OR caseload* OR caseload* OR (volume* ADJ3 outcome*) OR patient-volume* OR "volume of patients").ab,ti,kf.) AND (exp "Emergencies"/ OR traumatology/ OR "Emergency Treatment"/ OR exp "Emergency Medical Services"/ OR "Emergency Medicine"/ OR exp burns/ OR "Burn Units"/ OR exp "Fractures, Bone"/ OR exp accidents/ OR * "Wounds and Injuries"/ OR "Wounds, Nonpenetrating"/ OR exp "Wounds, Penetrating"/ OR "multiple trauma"/ OR exp "Craniocerebral Trauma"/ OR "Neck Injuries"/ OR "Extremities"/in OR "Athletic Injuries"/ OR exp "thorax"/in OR exp "Abdominal Injuries"/ OR exp "Trauma, Nervous System"/ OR ((emergen* ADJ3 (centre* OR center* OR ward* OR patient* OR admiss* OR admitt* OR department* OR room*)) OR trauma* OR neurotrauma* OR multitrauma* OR burn* OR fracture* OR accident* OR (acute* ADJ3 care) OR ((accidental OR blunt OR penetrating OR crush OR multiple OR head OR neck OR limb OR sport* OR athlet* OR thora* OR abdom* OR brain*) ADJ3 injur*) OR (ED ADJ3 patient*)).ab,ti,kf.) AND (exp "treatment outcome"/ OR exp hospitalization/ OR exp "mortality"/ OR mortality.xs. OR "Fatal Outcome"/ OR exp "survival"/ OR "length of stay"/ OR "convalescence"/ OR (outcome* OR (admiss* ADJ3 ("intensive care" OR icu OR icus OR hospital*)) OR mortalit* OR fatal* OR surviv* OR discharge* OR ((length OR duration* OR long* OR short*) ADJ6 stay) OR recover* OR convalescen* OR hospitalization* OR hospitalisation* OR ((treat* OR management* OR therap*) ADJ3 (fail* OR succes*))).ab,ti,kf.) NOT (exp animals/ NOT humans/) NOT (letter OR news OR comment OR editorial OR congresses OR abstracts).pt. AND english.la.

Cochrane CENTRAL

((((volume*) NEAR/3 (high OR low OR surg* OR hospital* OR critical-patient* OR severe-patient* OR trauma-patient* OR emergen*-patient* OR centre* OR center*)) OR caseload* OR case-load* OR (volume* NEAR/3 outcome*) OR patient-volume* OR 'volume of patients'):ab,ti) AND (((emergen* NEAR/3 (centre* OR center* OR ward* OR patient* OR admiss* OR admitt* OR department* OR room*)) OR trauma* OR neurotrauma* OR multitrauma* OR burn* OR fracture* OR accident* OR (acute* NEAR/3 care) OR ((accidental OR blunt OR penetrating OR crush OR multiple OR head OR neck OR limb OR sport* OR athlet* OR thora* OR abdom* OR brain*) NEAR/3 injur*) OR (ED NEAR/3 patient*)):ab,ti) AND ((outcome* OR (admiss* NEAR/3 ('intensive care' OR icu OR icus OR hospital*)) OR

mortalit* OR fatal* OR surviv* OR discharge* OR ((length OR duration* OR long* OR short*) NEAR/6 stay) OR recover* OR convalescen* OR hospitalization* OR hospitalisation* OR ((treat* OR management* OR therap*) NEAR/3 (fail* OR succes*))):ab,ti)

Web of science

TS=(((((volume*) NEAR/2 (high OR low OR surg* OR hospital* OR critical-patient* OR severe-patient* OR trauma-patient* OR emergen*-patient* OR centre* OR center*)) OR ((number-of) NEAR/1 (critical-patient* OR severe-patient* OR trauma-patient* OR emergen*-patient*)) OR ((high* OR low*) NEAR/1 exposure*) OR (size NEAR/2 (department* OR hospital* OR centre* OR center*)) OR caseload* OR case-load* OR (volume* NEAR/2 outcome*) OR patient-volume* OR "volume of patients")) AND (((emergen* NEAR/2 (centre* OR center* OR ward* OR patient* OR admiss* OR admitt* OR department* OR room*)) OR trauma* OR neurotrauma* OR multitrauma* OR burn* OR fracture* OR accident* OR (acute* NEAR/2 care) OR ((accidental OR blunt OR penetrating OR crush OR multiple OR head OR neck OR limb OR sport* OR athlet* OR thora* OR abdom* OR brain*) NEAR/2 injur*) OR (ED NEAR/2 patient*))) AND ((outcome* OR (admiss* NEAR/2 ("intensive care" OR icu OR icus OR hospital*)) OR mortalit* OR fatal* OR surviv* OR discharge* OR ((length OR duration* OR long* OR short*) NEAR/5 stay) OR recover* OR convalescen* OR hospitalization* OR hospitalisation* OR ((treat* OR management* OR therap*) NEAR/2 (fail* OR succes*))))) AND DT=(article) AND LA=(english)

Google Scholar

"high|low volume" emergency centres|centers|wards|patients|departments"|trauma|neurotrauma|multitrauma|burn|fractures |accidents|"acute care" outcome|"care|icu|hospital admission "|mortality|fatal|survival|discharge|"length*stay"|recovery|hos pitalization
